# The Choice of Healthcare Providers for Febrile Children after Introducing Non-professional Health Workers in a Malaria Endemic Area in Papua New Guinea

**DOI:** 10.3389/fpubh.2015.00275

**Published:** 2015-12-24

**Authors:** Takahiro Tsukahara, Seiritsu Ogura, Takuma Sugahara, Makoto Sekihara, Takuro Furusawa, Naoki Kondo, Toshihiro Mita, Hiroyoshi Endo, Francis Hombhanje

**Affiliations:** ^1^Department of International Affairs and Tropical Medicine, Tokyo Women’s Medical University, Tokyo, Japan; ^2^School of Economics, Hosei University Graduate School, Tokyo, Japan; ^3^Hosei Institute of Aging, Hosei University, Tokyo, Japan; ^4^Division of Southeast Asian Area Studies, Graduate School of Asian and African Area Studies, Kyoto University, Kyoto, Japan; ^5^Department of Health and Social Behavior, School of Public Health, The University of Tokyo, Tokyo, Japan; ^6^Department of Molecular and Cellular Parasitology, Juntendo University School of Medicine, Tokyo, Japan; ^7^Centre for Health Research and Diagnostics, Divine Word University, Madang, Papua New Guinea

**Keywords:** healthcare utilization, malaria, community health workers, health services demand, health-seeking behavior

## Abstract

**Background:**

Disease burden of malaria in Papua New Guinea (PNG) is the highest in Asia and the Pacific, and prompt access to effective drugs is the key strategy for controlling malaria. Despite the rapid economic growth, primary healthcare services have deteriorated in rural areas; the introduction of non-professional health workers [village health volunteers (VHVs)] is expected to improve antimalarial drug deliveries. Previous studies on PNG suggested that distance from households negatively affected the utilization of health services; however, price effect on healthcare demand decisions has not been explored. Empirical studies on household’s affordability as well as accessibility of healthcare services contribute to policy implications, such as efficient introduction of out-of-pocket costs and effective allocation of health facilities. Therefore, we investigate price responsiveness and other determinants of healthcare provider choice for febrile children in a malaria endemic rural area wherein VHVs were introduced.

**Methods:**

Cross-sectional surveys were conducted using a structured questionnaire distributed in a health center’s catchment area of East Sepik Province in the 2011/2012 rainy seasons. Caretakers were interviewed and data on fever episodes of their children in the preceding 2 weeks were collected. Mixed logit model was employed to estimate the determinants of healthcare provider choice.

**Results:**

Among 257 fever episodes reported, the main choices of healthcare providers were limited to self-care, VHV, and a health center. Direct cost and walking distance negatively affected the choice of a VHV and the health center. An increase of VHV’s direct cost or walking distance did not much affect predicted probability of the health center, but rather that of self-care, while drug availability and illness severity increased the choice probability of a VHV and the health center.

**Conclusion:**

The results suggest that the net healthcare demand increases with the introduction of a VHV. Allocations from the government’s budget are required to sustain VHV activities because the introduction of a small user fee could impede the utilization of a VHV. A large travel cost related to the choice of the health center suggests that resource allocation is required for the expansion of formal healthcare providers to adequately operate a referral system.

## Introduction

In Papua New Guinea (PNG), malaria is the leading cause of outpatient visits, the second leading cause of hospital admissions, and the fourth leading cause of hospital deaths (1). Children are the most vulnerable to malaria infection, illness, and death. The disease burden of malaria in PNG, which is the highest in Asia and the Pacific, was estimated to be 17.6 disability-adjusted life years per 1,000 capita per year in 2010.^1^ Along with improvements in vector control using a long-lasting insecticidal net, maximizing access to prompt quality diagnoses and appropriate treatment for malaria are key to reducing malaria-related morbidity and mortality, as proclaimed by the PNG National Health Plan 2011–2020 (2). Improving service delivery in rural regions wherein the majority (89%) of the population lives is the top health priority for achieving the key strategy for malaria control (2).

Papua New Guinea, a lower middle-income country, has shown rapid economic growth. PNG’s sustained positive gross domestic product (GDP) growth has been more than 5% since 2007 and is expected to reach 16% in 2015.^2^ Accordingly, the total health expenditure per capita at purchasing power parity (NCU per USD) has sharply increased from 67.2 in 2006 to 115.2 in 2011.^3^ However, expenditure for operational costs for rural health services was only 13.5% of the total health expenditures (2).

In urban PNG, people have numerous choices of health providers. Formal health services are provided by the government, church agencies, and a few private clinics and doctors. People are also able to seek treatment from informal sectors, such as traditional healers and neighbors. Western drugs are available from pharmacies, general shops, and street traders. However, the choice of healthcare providers is limited for rural majority because primary healthcare services in rural regions have deteriorated. The number of aid posts is insufficient and 29% of them did not operate in 2010 (1). A need exists to expand health services to the community level, particularly in areas wherein aid posts are not functioning (3).

Recently, non-governmental organizations have promoted and trained village health volunteers (VHVs) for improving mother and child health in PNG (4, 5). VHVs are key players for a strategy on a worldwide scale known as home-based management of malaria (HMM) to improve access to antimalarial drugs (6). This strategy aimed to provide prompt and adequate access to prepacked quality antimalarial drugs through a network of community resource individuals. Numerous interventional trials have confirmed the effectiveness of HMM (7–9); however, no study has estimated healthcare demand of non-professional community workers using a discrete choice model in routine clinical settings of malaria endemic regions.

Previous studies have indicated that determinants of the choices of healthcare providers can be supply-side factors (e.g., choice set of health services, price, time cost, and quality) (10–16) individual/household factors (e.g., income and/or assets, age, gender, education level, and type and severity of illness) (10, 11, 13–17), and social and geographical factors (e.g., ethnicity, infrastructure, and access) (13–15, 17). Previous epidemiological studies on PNG have suggested that the utilization of health facilities declined with distance (18, 19) and increased with male and infant patients (19) based on attendance records. However, these studies did not include socioeconomic factors. Only one report exists on the choice of healthcare providers in PNG for malaria treatment using a discrete choice model (20). However, the study did not consider commonly used determinants, such as cost for health facilities, economic status of a household, and health status of a patient. These exclusions make it difficult to compare the results with those of other countries and/or settings.

Therefore, this study uses discrete choice models to investigate households’ affordability and accessibility of healthcare providers for malaria treatment after the introduction of a VHV program^4^ and to contribute to policymaking for efficient introduction of out-of-pocket costs and effective allocation of health facilities.

## Materials and Methods

### Study Area and Target Population

We conducted our study in the rural region of East Sepik Province, PNG. The study site was a catchment area of a health center located 56 km by road from the provincial capital of Wewak. The area was situated in a lowland swamp along the coast and experienced hyperendemic malaria throughout the year. All caretakers with children aged <5 years who were living in villages in the study area were included as the target population. Among the 23 villages at the site, we excluded two villages from the target population because of the difficulty in accessing it by car and one more village as it was an uncooperative village. Consequently, 20 villages were involved in the study. According to the 2011 National Census (21), the total population of the 20 villages was 8,035 people in 1,415 households.

### Questionnaire Survey

We developed a structured questionnaire and conducted cross-sectional interview surveys in the rainy seasons (February 2011 and 2012) when malaria morbidity was relatively high in the study site. The survey was conducted only once in 18 villages and twice – 2011 and 2012 – in two villages closest to the health center. In the preceding 2 weeks of the interview, we collected data on the fever episodes of children, treatment choices, and caretaker and patient characteristics. Trained field assistants in the study area interviewed caretakers who were primarily their mothers, otherwise, adult household members who mainly took care of them, such as their fathers, aunts, or grandmothers. We also obtained information on the characteristics of the health facility from direct observation or interviews with health workers. In the study settings, patients with fever or a history of fever were diagnosed and treated as having malaria because of lack of diagnosis tools. Therefore, fever was considered as presumed malaria.

The caretaker’s choice of treatment was defined as “a treatment that a caretaker has selected as the initial action following the last onset of an acute fever in a child.” If caretakers give treatment only within their own households or simply monitor the child’s condition over time and do not provide medical treatment throughout the fever episode, we defined the treatment choice as “self-care.”

### Treatment Choice Alternatives in the Study Area

Caretakers were able to use informal health services within their own communities, such as self-care, seeking healthcare from neighbors, traditional health practitioners, VHVs, and/or general shops. We observed self-care treatments, such as taking a cold bath or herbal sweating bath, drinking herbal hot water, and/or using leftover western drugs. Caretakers rarely received western drugs from neighbors. Traditional health practitioners who treated patients with traditional herbal medicine and/or spiritual magic were present in most communities (17 out of 20). Each community assigned one VHV, who provided essential drugs, such as antimalarial drugs and antibiotics, for patients in the community after completing 1 month initial training. Unlike African and Asian countries, over-the-counter (OTC) drugs were not popular in the study area. Villagers did not have access to antimalarial drugs and antibiotics as OTC drugs. Acetaminophens were available in some general shops in several communities.

In rural PNG, health centers, sub-health centers, and aid posts provided formal health services. One health center and three aid posts were in the study and the surrounding areas. The health center was operated by the Catholic Church, with a nursing officer as the head and 11 health workers. The health center, a key health facility in the area, was open 24 h and covered a broad range of work, such as ambulatory practice, hospitalized care, delivery care, outreach public health activities, and supervision of VHVs. Antimalarial drugs and antibiotics were always available during the study period.

Antimalarial drugs and antibiotics were also available in three aid posts (two governmental facilities and one Catholic-run facility). In each, one health worker provided outpatient care during the daytime. Residents of the study site had to drive 50–100 km to the town of Wewak to gain access to healthcare services in a general hospital, their two clinics, or their three pharmacies. Neither public nor community-based health insurance was introduced in the study area.

### Discrete Choice Models

We apply the mixed logit model with random coefficients, which relaxes the independence of the irrelevant alternative assumption for discrete choice models with three or more alternatives. Following the notation described by Cameron and Trivedi (22), the utility associated with the alternative *j* selected by individual *i* is represented as

(1) $$\begin{array}{l} U_{\mathrm{ij}}=x_{ij}^{\prime }\beta_{i}+z_{i}^{\prime }\gamma_{ji}+\varepsilon_{\mathrm{ij}}\\ \text{ =}x_{ij}^{\prime }\beta +z_{i}^{\prime }\gamma_{j}+x_{ij}^{\prime }v_{i}+z_{i}^{\prime }w_{ji}+\varepsilon_{\mathrm{ij}} \end{array}$$

where *x_*ij*_* is an alternative-specific variable, *z_*i*_* is an individual-specific variable, and ϵ*_*ij*_* is the error term. In Eq. 1, β*_*i*_* = β + *v_*i*_* and γ*_*ji*_* = γ*_*i*_* + *w_*ji*_*, where *v_*i*_* and *w_*ji*_* are random terms of the coefficients. Individual *i* selects alternative *y_*i*_* from *m* alternatives to maximize the utility. Assuming that the error term ϵ*_*ij*_* distributes an extreme value distribution, the logit probability of the alternative *j* selected by individual *i* is given as

(2) $$P_{\mathrm{ij}}|v_{i},w_{ji}=\frac{\exp(x_{ij}^{\prime }\beta +z_{i}^{\prime }\gamma_{j}+x_{ij}^{\prime }v_{i}+z_{i}^{\prime }w_{ji})}{\sum_{l=1}^{J}\exp(x_{il}^{\prime }\beta +z_{i}^{\prime }\gamma_{l}+x_{ij}^{\prime }v_{i}+z_{i}^{\prime }w_{ji})},\;j=1,\ldots ,J.$$

The choice probability of the mixed logit model is the integral of the logit probability over a density function of β*_*i*_* and γ*_*ji*_*. The distribution for the density of the coefficients is commonly specified to be normal or lognormal. The lognormal distribution is used when the coefficient is expected to be the same sign for every individual (23). We use the simulation methods with 500 Halton draws to approximate the maximum log likelihood function. Arc elasticity was calculated as the percentage change of choice probability divided by the percent change of a unit of a variable. We calculated the percentage change from point 1 to 2 using the midpoint. Stata13 (StataCorp, TX, USA) and the user-written command *mixlogit* (24) were used for statistical analysis and model estimation.

### Outcome and Explanatory Variables

The outcome variable is caretakers’ initial choice for their child’s fever among the possible treatment choice alternatives. The explanatory variables are divided into two categories: alternative-specific and individual-specific variables. Alternative-specific variables represent direct costs, distance from house to the health providers, and drug availability of the health providers. Individual-specific variables represent a household’s assets, a patient’s gender, a patient’s age, severity of the illness as perceived by the caretaker, and the caretaker’s education. It is not practical for a mixed logit model to include a full set of random coefficients when the sample size is limited. Thus, we establish the model with random coefficients of four variables – direct costs, distance, drug availability, and assets – which we believe are more interesting from the perspective of affordability, accessibility, and availability of healthcare services.

Direct costs were defined as the sum of the examination fee, treatment costs, and travel costs. We estimated the direct cost for the choice alternatives that were not selected. Both treatment and travel costs in a household were 0 even when the other alternatives were selected. Generally, a patient was not charged for a VHV, but VHVs were allowed to demand a small amount. In the study period, eight VHVs charged Kina 1 (USD 0.48) for medical care. The travel costs for a VHV were also 0 because the treatment was done in the patient’s community or a neighboring community that was within walking distance. In contrast, a patient’s fee for the health center was Kina 1 (USD 0.48) in 2011 and Kina 3 (USD 1.43) in 2012. This fee included the examination, a prescription, drugs, and revisit costs. People living in communities near the health center or with inconvenience of public transportation were observed to travel on foot, resulting in free travel costs. A public transport vehicle to the health center was used for the other 14 communities. The one-way travel cost was Kina 3 (USD 1.43) or 7 (USD 3.33) depending on the distance from the health center. A one-way trip on a private transport vehicle to the health center cost K 100 (USD 47.62), but this use was not observed in this study. Since the coefficient of the direct cost is expected to have the negative sign for every individual, it was assumed to have lognormal distribution.

Distance to healthcare providers – a proxy for accessibility – is another important determinant of healthcare choice. We define the self-care distance in a household as 0. As previously mentioned, VHV was located within walking distance to a house. The distance from the house to VHV was calculated as a direct linear distance using both geographic positioning system (GPS) data that were manually collected using GPS devices and a digital map of the area (Pasco Satellite Ortho, Pasco Corporation, Japan). To calculate the distance to health providers beyond the normal walking distance at which people commonly use a public transport vehicle, we distinguish walking distance from transportation distance using a vehicle because the physical and time costs are viewed as different. Walking distance was calculated as the direct distance from a house to the nearest roadside. Transportation distance was measured as the road distance from the nearest roadside to a health facility. Since the coefficient of the distance is expected to have a negative sign for every individual, it was assumed to have a lognormal distribution.

We assume that the availability of antimalarial drugs is a proxy for the quality of healthcare providers because a better drug supply is expected to increase utilization by patients. In the study period, first-line antimalarial drugs included a combination of amodiaquine (AQ) or chloroquine (CQ) plus sulfadoxine-pyrimethamine (SP) for uncomplicated malaria and artesunate plus SP for severe malaria. We set the value of dummy variable for “drugs are available” equal to 1 when at least two drugs enabling the combination therapy were in stock. Otherwise, the dummy variable was set equal to 0, i.e., “drugs are not available.” In the health center, drugs were always available in the study period. However, we observed that some VHVs did not have drug stock. Drugs were to be supplied to the health center every 3 months in response to a VHV’s order. At their own expense, VHVs had to travel to the health center to collect drugs. Drug delivery from the provincial capital’s storage of medical goods to the health center was often delayed in the first quarter of the fiscal year, i.e., from January to March. In the study period, 14 communities were assigned as “drugs are available” and eight communities were categorized as “drugs are not available.” We did not have data on drug availability in a household. However, observations revealed no accessibility to OTC drugs in the area (as defined above), no possibility to have leftover SP, a single-dose therapy given at health facilities, and no use of combination drugs in observed self-care episodes. Therefore, we considered self-care in each household as “drugs are not available.” Since the coefficient of the drug availability is also expected to have the negative sign for every individual, it was assumed to have a lognormal distribution.

Income and expenditures in rural areas of developing countries fluctuate widely because many individuals are small farmers, and few workers earning wages exist. Therefore, using cross-sectional data to estimate annual income and/or expenditures is susceptible to error. In contrast, because assets are more precisely measured even in developing countries, they were used as a proxy variable for long-term economic status by constructing a linear index of asset ownership and housing characteristics using principle component analysis (25). Some individuals with high economic status may make more use of healthcare providers to keep higher health status. Others may make less use of healthcare service due to higher opportunity cost. Therefore, we assume the coefficient of the assets as having normal distribution.

Individual (i.e., patient) gender and age were included as explanatory variables because biological and cultural inequalities of gender and/or age may affect the demand for healthcare. Most econometric studies of healthcare demand included the health status of a patient as an explanatory variable because an individual with a lower health status is expected to seek more healthcare services. In econometric studies of healthcare demand, type of illness symptoms (10, 26, 27), severity of illness (13, 14), number of health days in the reference period (10, 13, 15), and duration of illness or inability to work (11, 28, 29) were used as proxies for health status. The number of health days and/or the duration of the illness or being unable to work may be endogenous to the choice of healthcare provider because the provider’s choice could influence these variables. To avoid this problem, we adopted perceived illness severity by caretakers at the onset of acute fever as a proxy for health status. We categorized the severity dummy as mild symptom equaled 0 and moderate or severe symptom equaled 1.

Caretaker education level is expected to increase healthcare demand because more educated individuals understand the benefits of healthcare in improving one’s health status. The educational structure system of PNG comprises 3 years of elementary, 6 years of primary, 2 years of lower secondary, and 2 years of upper secondary courses before tertiary (university and college) education. The study area had six primary schools and one lower secondary school. In this study, education level was defined by the total number of years of schooling.

### Ethical Clearance

Ethical clearance for the study was obtained from the Medical Research Advisory Committee of the Papua New Guinea National Department of Health (No. 09.26) and the Tokyo Women’s Medical University Ethical Committee (No. 1744). This study was conducted in accordance with the Declaration of Helsinki and the recommendations of those committees with written informed consent from all participants.

## Results

### Basic Statistics

Caretakers in 736 households with 1,012 children, or 86.5% of the target population, were interviewed. Out of this group, 257 children from 195 households were reported to have a fever episode. Among eight choice alternative sets selected, self-care was the most common (40.9% of the total episodes), followed by the VHV (31.5%) and the health center (20.6%). Some people asked for treatment at an aid post (2.7%), a pharmacy (2.0%), from neighbors (1.2%), our study team (0.8%), and a clinic (0.4%). We found that no one visited a hospital, a shop, or a traditional practitioner for the initial treatment. For further analysis on provider choice, we included the top three alternatives because they constituted 93.0% of the total episodes, and the characteristics of each of the other alternatives were too different to merge into one category.

Table 1 presents descriptive statistics of the outcome and explanatory variables used for discrete choice models. High correlation between the direct cost and transportation distance was found (0.98). We excluded the variable “transportation distance” from the final model estimation to avoid multicollinearity. The mean of the observed direct cost of the VHV (mean: 0.286; SD: 0.450) significantly differed from that of the health center (mean: 2.189; SD: 2.140; *t* = −7.76, df = 132, *p* < 0.0001) and of self-care (mean: 0; SD: 0; *t* = −6.53, df = 184, *p* < 0.0001).

**Table 1:** **Descriptive statistics**.

**Outcome variables**	**Obs**	**%**				

Self-care	105	43.93				
Village health volunteer (VHV)	81	33.89				
Health center	53	22.18				
Total	239	100.00				

**Explanatory variables**	**Obs**	**Mean**	**SD**	**Min**	**Max**	**Median**

Direct cost for self-care (Kina^a^)	239	0	0	0	0	0
Direct cost for VHV (Kina^a^)	239	0.319	0.465	0	2	0
Direct cost for health center (Kina^a^)	239	8.033	6.479	0	17	7
Walking distance to self-care (km)	239	0	0	0	0	0
Walking distance to VHV (km)	236	0.928	0.913	0	3.4	0.6
Walking distance to health center (km)	236	3.205	3.902	0	12.8	1.8
Transportation distance to self-care (km)^b^	239	0	0	0	0	0
Transportation distance to VHV (km)^b^	239	0	0	0	0	0
Transportation distance to health center (km)^b^	239	10.380	12.523	0	30.3	0
Antimalarial drug availability at self-care (no = 0/yes = 1)	239	0	0	0	0	0
Antimalarial drug availability at VHV (no = 0/yes = 1)	239	0.720	0.450	0	1	1
Antimalarial drug availability at health center (no = 0/yes = 1)	239	1	1	1	1	1
Gender of patient (female = 0/male = 1)	239	0.544	0.499	0	1	1
Age of patient (year)	239	2.096	1.373	0	4	2
Illness severity perceived by caretaker (mild = 0/moderate or severe = 1)	222	0.342	0.476	0	1	0
Education of caretaker (year)	232	6.586	2.635	0	14	6
Asset index	237	0.000	1.371	−1.699	6.355	−0.200

*^a^Kina 1 = USD 0.48 in 2011*.

*^b^Excluded from the final model*.

Since theoretical and empirical evidences have indicated returns to investment in education (30), the education level is expected to correlate with economic status. In our study, however, correlation between the education of caretakers and economic status of their household was not very high (0.15). Therefore, we included both in the model estimation.

**Figure 1:**
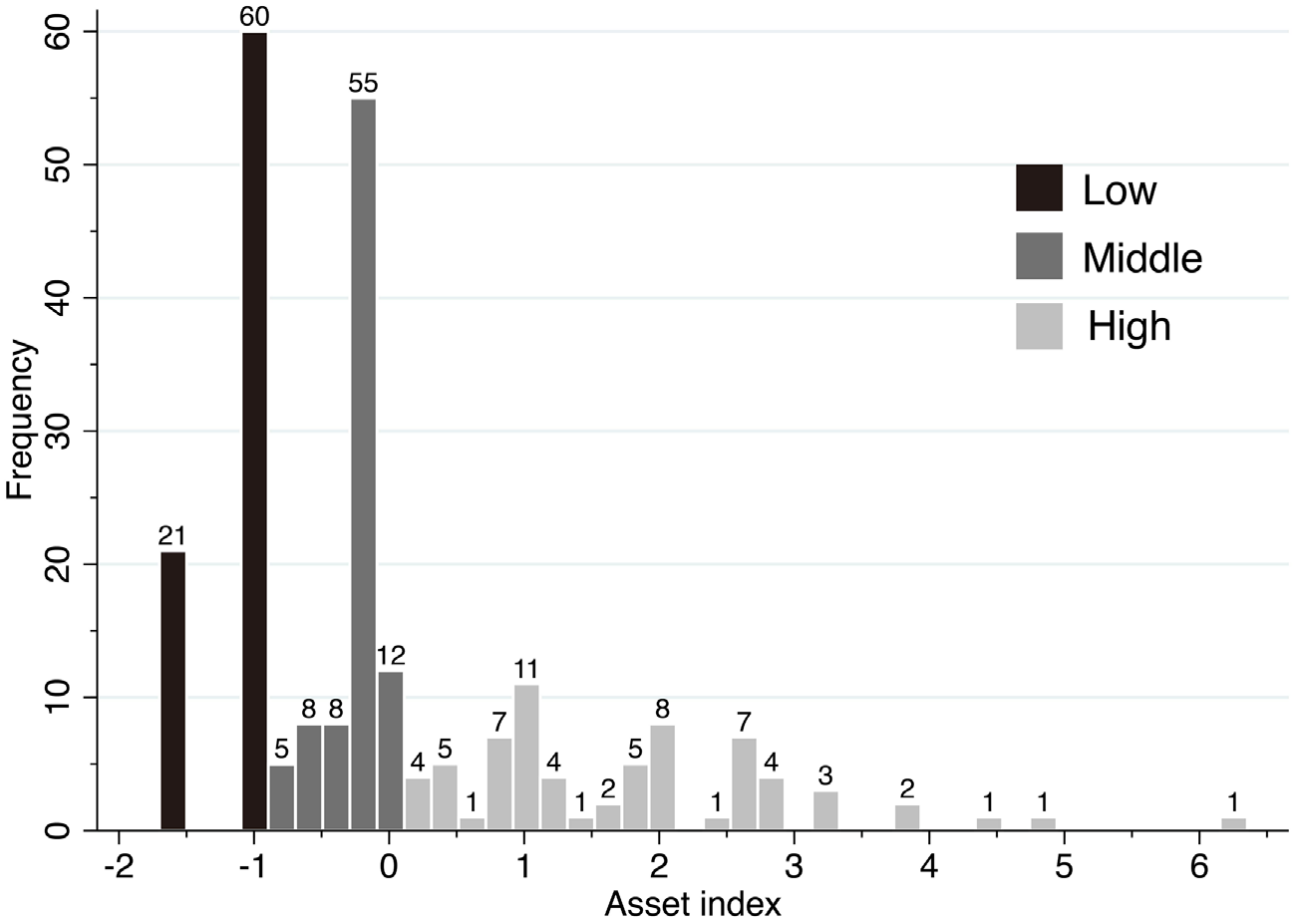
**Frequency distribution of asset indices**. Each bar chart represents frequencies in intervals of 0.2 of the asset index.

To estimate an asset index using principle component analysis to weigh the factor score of the first principal component, we selected seven dummy variables: own mobile phone (holding ratio; 81.8%), own radio or stereo (49.2%), own house with tin roof (24.6%), own generator (18.6%), own rainwater tank for drinking (12.0%), own western-style house (5.4%), and own car or outboard motorboat (4.2%). The eigenvalue of the first principal component was 1.88, and the ratio of valiance explained was 0.27. Since the distribution of the asset index was biased toward <0 with high frequencies at −1.6, −1.0, and −0.2 (Figure 1), we categorized the asset index into economic status tertiles: high, middle, and low. We included 210 observations to estimate the discrete choice models because of missing data on walking distance, illness severity, education level, and/or asset index. The observed choice ratios of self-care, VHV, and the health center were 0.429, 0.367, and 0.205, respectively.

### Model Estimation

We employed a mixed model to estimate healthcare providers for childhood fever with self-care as the base alternative. The average predicted probabilities of self-care, VHV, and the health center were 0.428, 0.366, and 0.207, respectively. As expected, direct cost and walking distance significantly decreased the choice probability of health providers, in contrast with a positive significant effect of drug availability on their choice probability (Table 2). The estimated SD of direct cost and walking distance was not significant and was small in magnitude. These results indicate that every individual is likely to have the same preference in response to a change in the value of these variables. The estimated SD of antimalarial drug availability was relatively high but not significant. This result may be attributable to the small sample size; however, 96% of individuals prefer “drugs are available” even if its SD was significant. These results reveal that the preferences for direct cost, walking distance, and drug availability regarding the choice of healthcare providers are not different among each person, which justifies the use of the lognormal distribution of the three variables.

**Table 2:** **Estimation results of the mixed logit model**.

		VHV	Health center
**MEAN**			
Direct cost (Kina; Kina 1 = USD 0.48)		−1.2977*** (0.408)			
Walking distance (km)		−0.6843*** (0.198)			
Antimalarial drug availability (no = 0/yes = 1)		2.0290*** (0.742)			
Gender of patient (female = 0/male = 1)		−1.0621** (0.514)	−1.8564** (0.862)
Age of patient (year)		−0.2646 (0.178)	−0.7626** (0.311)
Illness severity (mild = 0/moderate or severe = 1)		1.4926** (0.602)	3.4261*** (1.191)
Education of caretaker (year)		−0.2177** (0.102)	−0.1925 (0.214)
Economic status (low = base)	Middle	0.835 (0.672)	0.1147 (0.779)
	High	0.6924 (0.594)	−3.9198 (2.586)
Constant		0.9367 (0.981)	6.6108*** (2.508)
**SD**			
Direct cost		0.0039 (0.291)	
Walking distance		0.0039 (0.181)	
Antimalarial drug availability		1.1833 (1.358)	
Economic status	Middle	2.5479* (1.543)	0.0603 (1.964)
	High	0.0170 (2.680)	4.2573 (2.903)
Sample size		210	
Log likelihood		−148.98	
Pseudo R2		0.3280	

*Self-care is the base alternative. Numbers in parentheses are SEs*.

*Direct cost includes treatment cost and travel cost. Economic status is categorized into three based on asset index (see Figure 1)*.

*VHV, village health volunteer*.

***p < 0.01.

**p < 0.05.

*p < 0.1.

In contrast, the effect of economic status on the choice of healthcare providers was complex. Individuals with a middle economic status had a positive but not significant effect on the choice probability of VHV or the health center. Moreover, a significant difference in the individual preference of VHV choice was found: 37% of individuals disliked VHV and 63% favored VHV. In contrast, a high economic status had a negative but not significant effect on the choice of the health center. A difference in the individual preference for a health center was found but was not significant (82%: dislike; 18%: like).

Of the other variables with fixed coefficients, the sign of the effects on the choice of VHV was the same as that observed for the choice of the health center. Gender, age, and education had a negative effect, and severe illness had a positive effect. Besides the magnitude of the effect on education, the magnitude of the effect of the health center was larger than that of the VHV. These effects were significant except for the effect of age on the choice of the VHV and the effect of education on the choice of the health center.

### Choice Probabilities Regarding Changes in Direct Cost, Walking Distance, Drug Availability, or Illness Severity

**Figure 2:**
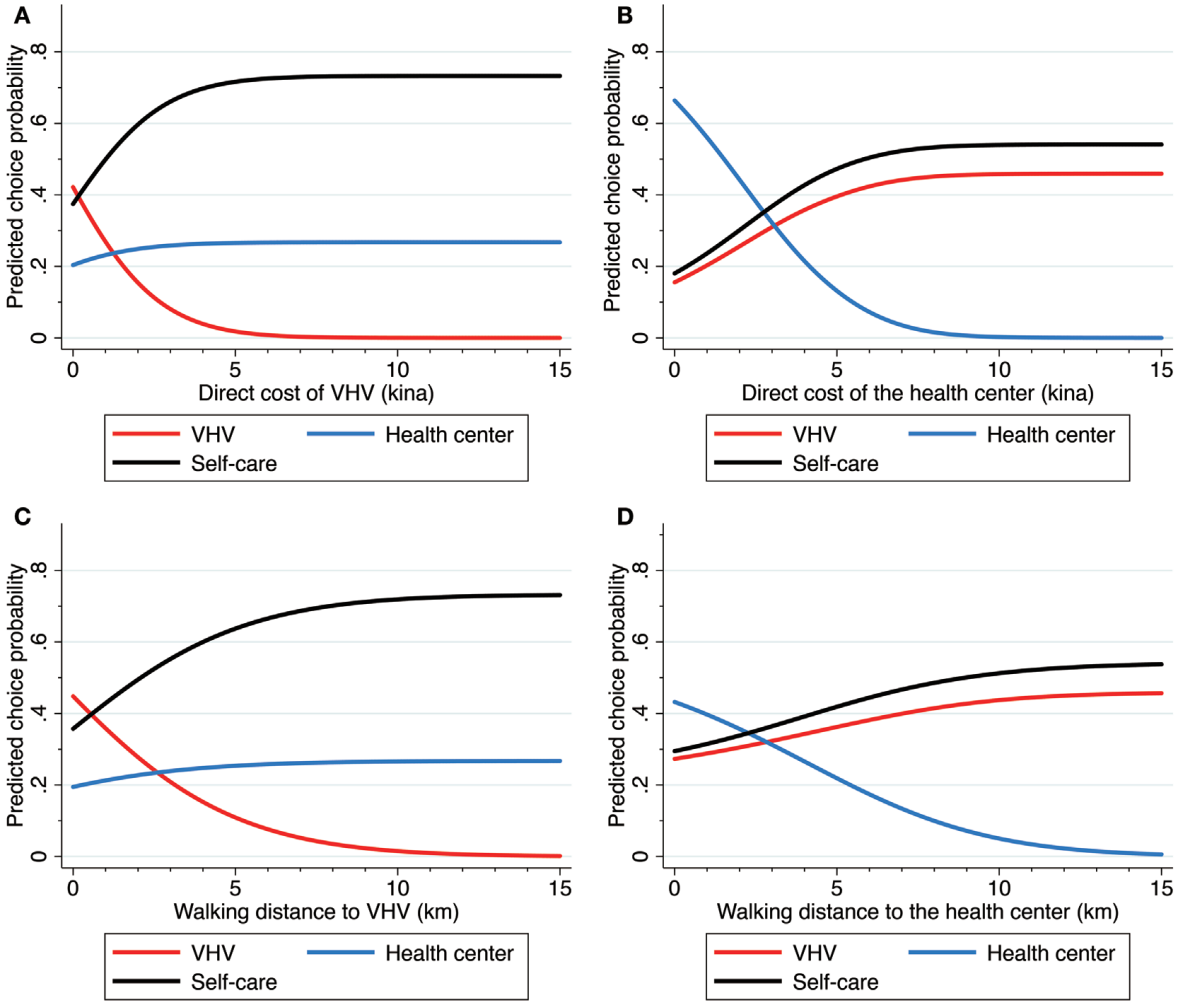
**(A)** shows the choice probabilities of the three alternatives for a change in the direct cost of village health volunteer (VHV). **(B)** shows the choice probabilities of the three alternatives for a change in the direct cost of the health center. **(C)** shows the choice probabilities of the three alternatives for a change in the walking distance to VHV. **(D)** shows the choice probabilities of the three alternatives for a change in the walking distance to the health center. Direct cost includes treatment cost and travel cost. The unit of direct cost is in Papua New Guinean Kina (Kina 1 = USD 0.48 in 2011).

We calculated the average predicted probabilities of the choice alternatives regarding changes in the direct cost or walking distance of a VHV or the health center (Figures 2A–D). With no direct cost of the VHV, the probability of the VHV choice slightly increased to 0.422 compared with the average choice probability calculated from the sample enumeration (0.366). The probabilities rapidly declined to 0.269, 0.154, and 0.081 as the direct cost changed by Kina 1 (USD 0.48), 2 and 3, respectively (Figure 2A). When its direct cost was more than Kina 5.7, the probability declined to <0.01. As the direct cost of VHV increased, the choice probability of self-care increased strikingly from 0.374 to 0.733. Meanwhile, an increase in the VHV direct cost slightly increased the choice probability of the health center from 0.204 to 0.267. Then, we calculated price arc elasticities given a change in the VHV direct cost from 0 to Kina 5 (Table 3). The own arc elasticity of VHV demand was −1.836, and the cross arc elasticities of self-care and the health center were 0.627 and 0.264, respectively.

**Table 3:** **Arc elasticities of healthcare provider choice**.

Variable changed	Arc elasticity		
	Self-care	VHV	Health center
Direct cost of VHV (0–Kina 5)	0.627	−1.836	0.264
Direct cost of health center (0–Kina 5)	0.895	0.872	−1.339
Walking distance of VHV (0–5 km)	0.563	−1.217	0.263
Walking distance of health center (0–5 km)	0.348	0.281	−0.655

*Values in parentheses are a unit of a variable changed. Direct cost includes treatment cost and travel cost. Kina 1 = USD 0.48 in 2011*.

*VHV, village health volunteer*.

With no direct cost related to the health center, the probability of choosing the health center dramatically increased to 0.664 from the average choice probability calculated from the sample enumeration (0.207). The probabilities rapidly declined to 0.442, 0.322, 0.215, and 0.132 as the direct cost changed by Kina 2, 3, 4, and 5, respectively (Figure 2B). The probability declined to <0.01 when its direct cost was higher than Kina 8.5. Given an increase in the direct cost of the health center, both the choice probability of self-care and that of VHV considerably increased from 0.181 to 0.541 and from 0.155 to 0.459, respectively. Thus, given a change in the direct cost of the health center from 0 to Kina 5, the own arc elasticity was −1.339 and the cross arc elasticities of self-care and VHV were 0.895 and 0.872, respectively (Table 3).

As the walking distance to VHV increased, the choice probability of the VHV declined (Figure 2C). The probability was 0.448 with no walking distance and decreased to 0.358 at 1 km, 0.278 at 2 km, 0.209 at 3 km, and 0.109 at 5 km. The probability was <0.01 for distances longer than 10.9 km. Given an increase in the walking distance to the VHV, the choice probability of self-care substantially increased from 0.357 to 0.733, but the choice probability of the health center slightly increased from 0.195 to 0.267. Consequently, given a change in the walking distance from 0 to 5 km, the own arc elasticity of VHV was −1.217 and the cross arc elasticities of self-care and the health center were 0.563 and 0.263, respectively (Table 3).

As shown in Figure 2D, the choice probability of the health center declined as the walking distance from the health center increased. This probability was 0.432 with no distance and decreased to 0.357 at 2 km, 0.219 at 5 km, and 0.100 at 8 km. The probability declined to <0.01 for distances longer than 13.8 km. As the walking distance to the health center increased, the choice probability of self-care and VHV increased from 0.295 to 0.541 and from 0.273 to 0.459, respectively. Given a change in the walking distance from 0 to 5 km, the own arc elasticity of the health center was −0.655 and the cross arc elasticities of self-care and VHV were 0.348 and 0.281, respectively (Table 3).

Given a sufficiently large value of direct cost of VHV or walking distance of VHV, we can consider that all VHVs are neither affordable nor accessible. At that time, the average choice probabilities of self-care and the health center were 0.733 and 0.267, respectively (Figures 2A,C).

We calculated the average choice probabilities of a change in two dummy variables: antimalarial drug availability and illness severity (Table 4). The probability of VHV choice slightly increased to 0.427 when drugs were available at all VHVs. Instead, the choice probability of VHV markedly decreased to 0.188 when no drugs were available at all VHVs. Given a change in the drug availability of VHV, the choice probability of self-care and the health center increased from 0.390 to 0.575 and from 0.183 to 0.237, respectively. When no drugs were available at the health center, the choice probability of the health center decreased to 0.100, and that of self-care and VHV increased to 0.485 and 0.415, respectively.

**Table 4:** **Predicted probabilities of healthcare provider choice regarding a change of antimalarial drug availability or illness severity**.

	Predicted probability						
	Self-care	VHV	Health center
Average (sample enumeration)	0.428	0.366	0.207
**Antimalarial drug availability**			
Not available at all the VHVs	0.575	0.188	0.237
Available at all the VHVs	0.390	0.427	0.183
Not available at the health center	0.485	0.415	0.100
**Illness severity**			
Mild for all the patients	0.510	0.334	0.156
Moderate or severe for all the patients	0.265	0.425	0.311

*VHV, village health volunteer*.

When all patients had a mild symptom, the choice probabilities of self-care, VHV, and the health center were 0.510, 0.334, and 0.156, respectively. In contrast, the choice probabilities of self-care, VHV, and the health center changed to 0.265, 0.425, and 0.311, respectively, when all individuals were moderate or severe patients.

## Discussion

This study had the following four main findings related to choosing healthcare providers for febrile children. (a) Direct cost was a negative significant determinant of the choice of both a VHV and the health center, (b) the choice probability of both a VHV and the health center significantly declined as the walking distance from them increased, (c) drug availability significantly increased the probability of VHV utilization, and (d) illness severity was another significant determinant of the choice of both a VHV and the health center.

Previous studies have used subgroup analysis to indicate that the price elasticity of healthcare demand for children is higher than that for adults (11, 16, 27). Some studies have also indicated high price elasticities of demand for child healthcare (11, 16), a finding that is compatible with our finding of the negative effect of direct cost on demand for healthcare for febrile children.

Our study using adequate econometric model with well-controlled variables confirmed the importance of the effect of walking distance on healthcare demand suggested by previous studies in PNG (18–20). We also show that the effect of direct cost as well as walking distance on utilization of VHV is larger than that of the health center based on the magnitude of own elasticities (Table 3).

Regarding an increase in the VHV user fee, demand for the health center does not increase much compared with the significant increase in demand for self-care (Figure 2A). Instead, given an increase in the direct cost of the health center, the cross elasticity of VHV is similar to that of self-care (Figure 2B, Table 3). In addition, a similar substitutional relationship was found between self-care choice and VHV use when walking distance changed (Figures 2C,D). These results suggest that a higher substitutability exists between VHV and self-care than between VHV and the health center given a change in the direct cost or walking distance. Thus, the introduction of a VHV can draw substantial demand shift from self-care to VHV, but lead to slight reduction of the health center demand. We estimated VHV demand under the condition of unaffordability or inaccessibility, which may be considered as approximative conditions of the pre-introduction of VHV. In this case, the predicted probabilities of self-care and the health center are estimated to decrease from 0.733 to 0.428 and from 0.267 to 0.207 before and after the introduction of a VHV. Consequently, we speculate that the introduction of a VHV leads to incremental healthcare demand, which is equivalent to the decrease in the self-care probability (0.305), by drawing on the demand shift from self-care to VHV.

Caretakers with a child suffering from a more severe illness are expected to seek higher quality health services. As expected, the more severe the condition of the child as perceived by caretakers, the more frequently they visited healthcare providers, a finding that is in line with that of previous studies (13, 14). The anticipated association between drug availability from the VHV and utilization of a VHV concurs with previous reports (12, 31).

A previous study in Kenya reported negative effect of antimalarial drug availability on health demand (32). They speculated that the lack of antimalarial drugs may be evidence of high demand of health facilities. Another empirical study found positive association between drug availability and health demand (33). Our study suggests that drug availability positively impacts the choice of healthcare provider.

If healthcare provider choice is wealth elastic, rich caretakers are expected to be more likely to use the health center because its average direct cost is the highest among the three alternatives. In contrast, the study result indicates that higher economic status decreases the use of the health center. This phenomenon occurs partly because the significant determinant of the choice of the health could not be the direct cost but time cost for individuals with higher economic status. Additionally, children with higher economic status may have better general health status (34), suggesting that their caretakers are not motivated to use healthcare that has a high time cost.

A caretaker with a higher education was more likely to treat a child with self-care. This result is in accordance with that reported by Levin et al. (35) – who believe that with regard to self-care, educated caretakers are more confident than less educated ones. Another possibility is that the positive association between parental educational level and health status of children (34, 36) may relate to a lower need to use health facilities by children of highly educated parents.

In some developing countries, the female gender has fewer possibilities of obtaining health services, which is considered to be due to a reflection of gender inequalities (37–39). Similarly, qualitative research on PNG reveals that adult females are less likely to access health services because of gender discrimination (40). In contrast to these studies, our result that caretakers made fewer visits to health facilities for male children is in accordance with the result from a study on outpatient demand (41). A study in PNG using household expenditure data reported gender disparity in children, i.e., a preference for boys (42). Insufficient care for female children may lead to a lower health status of female children, including high risk of malnutrition, which is reported to occur in PNG (43). Therefore, caretakers may understand that a female’s vulnerability is derived from a lower health status, causing greater use of healthcare providers when female children are ill, as speculated in a previous study (41).

Direct cost of a VHV equaled to its user treatment fee because its travel cost was free in the study area. As shown in Figure 2A, even a small amount of use fee, such as USD 1, can considerably decrease the chance of VHV utilization. Since the introduction of a user fee seems questionable, allocations from the government’s budget are required to sustain VHV activities. We believe that an appropriate drug supply is significant to the choice of VHV. In addition, constructing a referral system is important for the management of severe illness as well as treatment for prevention of *vivax* malaria – i.e., administration of primaquine, which is prohibited for the VHV in PNG. The government should attempt to expand referral health facilities to reduce travel cost for communities far from an existing health center to overcome the barrier of access to a referral health facility. Reactivating closed aid posts could be a solution.

Our study has several limitations. First, the external validity of this study was limited because it was conducted in a catchment area of a health center with a limited number of illness episodes. The results cannot be generalized to other areas in which VHVs were introduced. The second problem is an omitted variable. Transportation distance, a proxy of the time cost of transportation, could not be included in the model because of the high correlation between direct cost and transportation distance. Considering the fact that a vital portion of the direct cost of the health center is transportation, the estimated high price sensitivity regarding the choice of the health center is overestimated, and we should interpret the value as the effect of a mix of the price and the time cost of transportation. Third, we must consider that caretakers may not receive proper information on drug availability, although we used actual drug availability data of the VHV and assumed that caretakers had information on the true availability of drugs. This uncertainty of the information that caretakers did or did not have could bias the results. Fourth, self-reported episodes could be subject to a recall bias, though we used shorter 2-week recall period compared to a 4-week recall period used in the previous study in PNG (20).

Despite these limitations, to our knowledge, this is the first study to explore households’ affordability as well as accessibility of healthcare services in PNG and develop a discrete choice model including an alternative for a VHV, a non-professional health worker for distributing essential drugs. Unlike many African and Southeast Asian countries where OTC drugs for fever treatment are commonly available, possible alternatives were limited in our setting, permitting a simple estimation of models and an interpretation of the results. We hope that further investigation will be performed under the setting with non-professional health workers in other areas with adequate econometric models.

## Author Contributions

TT conceived the study, designed the study, conducted the data collection, performed the econometric analysis, wrote the first draft of the manuscript, and completed the final version. HE participated in the study design. MS, TF, NK, TM, and FH ­participated in the study design and conducted the data collection. SO and TS interpreted the data and revised the draft. All authors read and approved the final draft.

## Conflict of Interest Statement

The authors declare that the research was conducted in the absence of any commercial or financial relationships that could be construed as a potential conflict of interest. The funders of this study played no direct role in its design or execution.

## Footnotes

^1^http://vizhub.healthdata.org/irank/heat.php

^2^http://data.worldbank.org/indicator/NY.GDP.MKTP.KD.ZG/countries/

^3^http://hiip.wpro.who.int/portal/DataAnalytics/AccessDataBySource.aspx

^4^Part of the results using the same data set has been presented at the ninth annual meeting of the Japanese Health Economics Association on September 6, 2014 (in Japanese). A preliminary abstract written in Japanese is available at http://www.ihep.jp/jhea/convention/list.html.
